# Outcomes following cardiac resynchronisation therapy in older people

**DOI:** 10.1093/ageing/afad222

**Published:** 2023-11-30

**Authors:** Nawaz Z Safdar, Stephe Kamalathasan, Ankit Gupta, Joshua Wren, Rory Bird, Dorothy Papp, Rebecca Latto, Ali Ahmed, Victoria Palin, John Gierula, Klaus K Witte, Sam Straw

**Affiliations:** School of Medicine, Faculty of Medicine and Health, University of Leeds, Leeds, UK; Department of Cardiology, Bradford Teaching Hospitals NHS Trust, Bradford, UK; School of Medicine, Faculty of Medicine and Health, University of Leeds, Leeds, UK; Department of Cardiorespiratory Medicine, Leeds Teaching Hospitals NHS Trust, Leeds, UK; School of Medicine, Faculty of Medicine and Health, University of Leeds, Leeds, UK; School of Medicine, Faculty of Medicine and Health, University of Leeds, Leeds, UK; School of Medicine, Faculty of Medicine and Health, University of Leeds, Leeds, UK; School of Medicine, Faculty of Medicine and Health, University of Leeds, Leeds, UK; Department of Cardiorespiratory Medicine, Leeds Teaching Hospitals NHS Trust, Leeds, UK; School of Medicine, Faculty of Medicine and Health, University of Leeds, Leeds, UK; Medicine Clinic 1, RWTH Aachen University, Aachen, Germany; School of Medicine, Faculty of Medicine and Health, University of Leeds, Leeds, UK

**Keywords:** response, outcomes, heart failure, cardiac resynchronization therapy, older people

## Abstract

**Introduction:**

Older patients may be less likely to receive cardiac resynchronisation therapy (CRT) for the management of heart failure. We aimed to describe the differences in clinical response, complications, and subsequent outcomes following CRT implantation compared to younger patients.

**Methods:**

We conducted a retrospective cohort study of unselected, consecutive patients implanted with CRT devices between March 2008 and July 2017. We recorded complications, symptomatic and echocardiographic response, hospitalisation for heart failure, and all-cause mortality comparing patients aged <70, 70–79 and ≥ 80 years.

**Results:**

Five hundred and seventy-four patients (median age 76 years [interquartile range 68–81], 73.3% male) received CRT. At baseline, patients aged ≥80 years had worse symptoms, were more likely to have co-morbidities, and less likely to be receiving comprehensive medical therapy, although left ventricular function was similar. Older patients were less likely to receive CRT-defibrillators compared to CRT-pacemakers. Complications were infrequent and not more common in older patients. Age was not a predictor of symptomatic or echocardiographic response to CRT (67.2%, 71.2% and 62.6% responders in patients aged <70, 70–79 and ≥ 80 years, respectively; *P* = 0.43), and time to first heart failure hospitalisation was similar across age groups (*P* = 0.28). Ten-year survival was lower for older patients (49.9%, 23.9% and 6.8% in patients aged <70, 70–79 and ≥ 80 years, respectively; *P* < 0.001).

**Conclusions:**

The benefits of CRT on symptoms and left ventricular function were not different in older patients despite a greater burden of co-morbidities and less optimal medical therapy. These findings support the use of CRT in an ageing population.

## Key Points

- Symptomatic or echocardiographic improvements following implantation with cardiac resynchronisation therapy (CRT) were consistent in older (≥80 years) people.
- At baseline, older people had worse symptomatic status and were more likely to have co-morbidities.
- On an individual level, the absence of improvement following cardiac resynchronisation therapy does not always represent a lack of treatment effect.

## Introduction

### Background

Chronic heart failure is a growing public health problem primarily affecting older individuals [1]. Although its incidence is decreasing in part due to the improved management of cardiovascular diseases including ischaemic heart disease and hypertension, the background of an ageing population means the overall prevalence is increasing [2]. For those who have heart failure with a reduced ejection fraction (HFrEF), cardiac electrical and mechanical dysynchrony can have the effect of making an impaired left ventricle even less effective. For this reason, cardiac resynchronisation therapy (CRT), a specialised form of pacemaker, is recommended for individuals with persistent symptoms of heart failure, left ventricular systolic dysfunction and a broad QRS complex on electrocardiogram (ECG).

CRT improves symptoms, reduces hospitalisation risk and extends life [3–5]. However, the relevant trials were conducted primarily in younger populations and it is feasible that the benefits of CRT are attenuated in older individuals who are more likely to have competing risks of non-cardiovascular morbidity [6]. Furthermore, older patients are perceived to be at an increased risk of implant related complications, adding to the risk of de-selection for CRT even when indicated [7].

### Aims

We aimed to explore whether there were age-related differences in clinical and echocardiographic response, complication rates and patient-orientated outcomes in a real-world cohort of people with heart failure receiving CRT devices.

## Methods

### Study design

We conducted a retrospective cohort study at a single tertiary centre in the United Kingdom. Consecutive patients undergoing implantation of CRT with or without a defibrillator for the management of HFrEF between March 2008 and July 2017 were eligible for inclusion. The institutional review board approved the study (IRB #9434 30/07/2021) and, in view of the retrospective nature, individual patient consent was waived as appropriate data protection safeguards were in place.

### Study procedures

We collected patient demographics (age, sex and ethnicity) and co-morbidities (ischaemic heart disease, hypertension, diabetes mellitus, atrial fibrillation, prior stroke, chronic kidney disease [stage IV or V] and chronic obstructive pulmonary disease). We recorded medications at the time of the implant and reported doses of beta-blockers, angiotensin-converting-enzyme inhibitors (ACEi) (or angiotensin II receptor blockers [ARB]) and loop diuretics as equivalent doses relative to the maximum licensed doses of bisoprolol, ramipril and furosemide as described previously [8].

We also collected laboratory tests including haemoglobin, creatinine, albumin, glycosylated haemoglobin (HbA1c) and *N*-terminal pro B-type natriuretic peptide (NT-proBNP). A standard 12-lead electrocardiogram at a baseline of 25 mm/s documented the PR interval, QRS duration, and morphology. Ventricular conduction delay was classified as either left bundle branch block, right bundle branch block or non-specific interventricular block. Two-dimensional transthoracic echocardiography was done according to recommendations in place at the time [9]. Where endocardial border definition allowed, left ventricular ejection fraction (LVEF) was determined by Simpson’s biplane method and where this was not possible a visual estimation of LVEF was provided with LV function graded as mildly, moderately or severely impaired.

### Definition of response

Response to treatment in a cohort, especially longevity gain, for a chronic, incurable condition is difficult to determine in the absence of an alternatively treated comparator group. However, our aim was to compare outcomes across age groups. Hence, for the purposes of this manuscript, we defined response to CRT as either symptomatic improvement, defined as ≥1 point improvement in New York Heart Association (NYHA) classification [10], or an improvement in LV systolic function, defined as ≥10% improvement of LVEF [11] from baseline. Where measurement or visual estimation of LVEF was not provided, an improvement of ≥1 subjective categories (mild, moderate, severe) was regarded as an echocardiographic response.

### Ascertainment of outcomes

**Figure 1:**
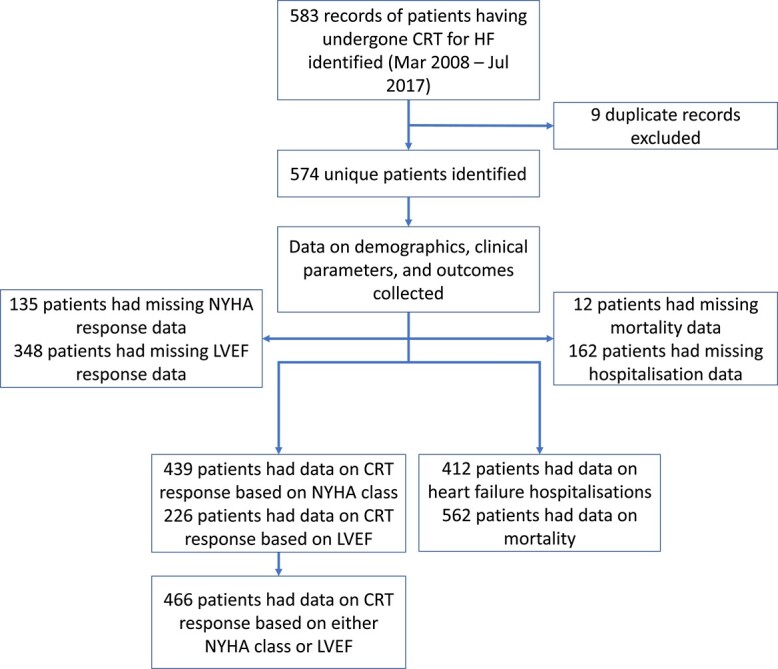
Flowchart depicting the patient characteristics available at each stage of the study.

Data relating to complications following CRT implantation, all-cause mortality and hospitalisation for heart failure were collected from the Leeds Patient Pathway Manager Plus electronic health record (Figure 1).

### Statistical analyses

Normality of distribution was assessed visually by distribution plots and confirmed using skewness testing. Continuous (parametric) data are presented as means ± standard deviations (SD), and non-parametric data are presented as median (interquartile range [IQR]). Discrete variables are presented as numbers (percentages). Statistical comparison was performed using the Student’s t-test or analysis of covariance for parametric data, the Mann–Whitney U- or Kruskal–Wallis H-tests for non-parametric data, and the Pearson χ^2^ test for discrete data. Kaplan–Meier plots were used to plot survival and groups were compared using the Log-rank test. Odds ratios (OR), 95% confidence intervals (95% CI) and simple linear regressions were used to determine predictors of CRT response. No imputation was made for missing data. Statistical analyses were done using Stata (StataCorp. 2019. Stata Statistical Software: Release 16. College Station, TX: StataCorp LLC.), with OriginPro 2020 (Origin(Pro), Version 2020. OriginLab Corporation, Northampton, MA, USA) used for illustrations. Statistical significance was defined as a *P*-value of <0.05 and all tests were two-sided.

## Results

### Patients

Between March 2008 and July 2017, a total of 574 individuals were implanted with CRT devices for the management of HFrEF. The median age was 76 years (68–81) and 421 (73.3%) patients were male. The age distribution of the cohort is depicted in Supplementary Figure 1. To compare differences in characteristics and outcomes according to age, we calculated tertiles where the lower tertile was 72 years and the upper tertile was 79 years. For simplicity, we divided patients into three age categories which were < 70 (*n* = 158, 27.5%), 70–79 (*n* = 243, 42.3%) and ≥ 80 years (*n* = 173, 30.1%). Descriptive clinical characteristics divided by age groups are summarised in Table 1. Older patients were more likely to have worse symptomatic status, with more having NYHA class III or IV symptoms (57.3% in <70 years versus 68.7% in 70–79 years versus 72.2% in ≥80 years; *P* = 0.011). Although the distribution of diabetes and hypertension was similar, older individuals were more likely to have a history of ischaemic heart disease (*P* < 0.001), atrial fibrillation (*P* = 0.026) and chronic kidney disease (*P* = 0.030). Conversely, they were less likely to have a history of prior stroke (*P* = 0.046).

**Table 1:** Characteristics of the cohort split by age groups (<70, 70–79 and ≥ 80 years)

	All patients	<70 years	70–79 years	≥80 years	*P*-value
*Demographics*					
Age (years; median [IQR])	76 (68–81)	63 (57–66)^*^^*^	76 (73–77)^*^^*^	83 (81–87)^*^^*^	<0.001
Male sex (n [%])	421 (73.3)	110 (69.6)	175 (72.0)	136 (78.6)	0.15
Caucasian ethnicity (n [%])	503 (95.1)	140 (93.3)	213 (95.9)	150 (96.1)	0.36
NYHA class III or IV (n [%])	379 (66.6)	90 (57.3)^*^	167 (68.7)^*^	122 (72.2)^*^	0.011
*Co-morbidities*					
IHD (n [%])	335 (59.7)	73 (47.1)^*^^*^	147 (61.8)^*^^*^	115 (68.5)^*^^*^	<0.001
Hypertension (n [%])	119 (24.1)	30 (21.1)	49 (23.6)	40 (28.0)	0.39
Diabetes (n [%])	172 (30.4)	51 (32.7)	79 (32.6)	42 (25.0)	0.19
AF (n [%])	190 (35.1)	40 (26.3)^*^	87 (37.8)^*^	63 (39.6)^*^	0.026
Stroke (n [%])	21 (4.2)	11 (7.6)^*^	7 (3.4)^*^	3 (2.1)^*^	0.046
CKD (stage IV or V) (n [%])	88 (16.8)	17 (11.4)^*^	36 (16.3)^*^	35 (22.7)^*^	0.030
COPD (n [%])	61 (10.7)	12 (7.7)	34 (14.0)	15 (8.9)	0.11
*Blood tests*					
Haemoglobin (g/L)	131.2 ± 22.2	133.7 ± 19.1	131.7 ± 26.8	128.2 ± 17.4	0.093
Creatinine (μmol/L)	105 (85–135)	93 (77.5–114)^*^^*^	108 (84–132)^*^^*^	118 (95–149)^*^^*^	<0.001
Albumin (g/L)	42 (39–45)	43 (25–53)^*^	42 (26–50)^*^	41 (26–50)^*^	0.0015
HbA1c (mmol/mol)	49 (41–59)	48 (41–65)	50 (43–60)	47.5 (40–57)	0.52
NT-pro-BNP (ng/L)	2,063 (720–5,038)	904 (516–2967.5)	2,013 (788–6,120)	2,473 (1,003–6,994)	0.13
*Electrocardiogram findings*					
Heart rate (beats/min)	66.8 ± 16.6	67.9 ± 16.5	65.6 ± 17.3	67.3 ± 15.7	0.36
PR interval (ms)	184 (164–220)	177 (160–200)^*^^*^	189 (170–224)^*^^*^	195 (175–233)^*^^*^	<0.001
QRS interval (ms)	153.6 ± 23.9	152.9 ± 23.9	151.9 ± 24.9	156.7 ± 22.0	0.25
Left bundle branch block (n [%])	424 (75.3)	119 (76.8)	181 (75.1)	124 (74.3)	0.87
*Echocardiogram findings*					
LVEF % (median [IQR])	25 (23–35)	27 (25–34.8)	25 (20–34)	25 (25–35)	0.23
LVEDd (mm)	58.8 ± 8.6	60.5 ± 8.5	59.6 ± 8.5	56.2 ± 8.3	<0.001
LVESd (mm)	48.8 ± 10.6	50.3 ± 10.7^*^	49.7 ± 11.4^*^	45.7 ± 8.6^*^	0.046
*Pharmacotherapy*					
Beta-blocker (n [%])	524 (92.4)	146 (94.8)	225 (92.6)	153 (90.0)	0.26
Bisoprolol dose (mg)	5.2 ± 3.4	6.2 ± 3.5^*^^*^	5.2 ± 3.3^*^^*^	4.1 ± 2.9^*^^*^	<0.001
ACEi (n [%])	343 (60.4)	100 (64.5)	148 (60.9)	95 (55.9)	0.28
Ramipril dose (mg)	6.2 ± 3.3	6.8 ± 3.3^*^	6.3 ± 3.2^*^	5.3 ± 3.4^*^	0.0080
ARB (n [%])	143 (25.2)	41 (26.5)	63 (25.9)	39 (22.9)	0.72
Sacubitril/Valsartan (Entresto) (n [%])	1 (0.2)	1 (0.7)	0 (0.0)	0 (0.0)	n/a
ACEi/ARB/Entresto (n [%])	463 (83.1)	140 (90.9)^*^^*^	199 (84.0)^*^^*^	124 (74.7)^*^^*^	<0.001
Aldosterone antagonist (n [%])	261 (46.7)	87 (56.9)^*^	104 (43.5)^*^	70 (41.9)^*^	0.012
Loop diuretic (n [%])	414 (72.8)	106 (67.9)	178 (73.3)	130 (76.5)	0.22
Furosemide dose (mg)	71.4 ± 50.5	78.4 ± 57.3	72.5 ± 49.9	64.0 ± 44.4	0.16

^*^≤0.05

^**^≤0.001

Mann–Whitney U test to compare non-parametric data

Pearson χ2 test to compare discrete data

NYHA, New York Heart Association; IHD, ischaemic heart disease; AF, atrial fibrillation; CKD, chronic kidney disease; COPD, chronic obstructive pulmonary disease; NT-pro-BNP, N-terminal pro B-type natriuretic peptide; LVEF, left-ventricular ejection fraction; LVEDd, left ventricular end-diastolic diameter; LVESd, left ventricular end-systolic diameter; ACEi, Angiotensin-converting-enzyme inhibitor; ARB, Angiotensin-receptor-blocker

Between age categories, there were no differences in the QRS duration (152.9 ± 23.9 in <70 years versus 151.9 ± 24.9 in 70–79 years versus 156.7 ± 22.0 in ≥80 years; *P* = 0.25) or the proportion of patients who had left bundle branch block morphology (76.8% in <70 years versus 75.1% in 70–79 years versus 74.3% in ≥80 years; *P* = 0.87), although older patients had a longer PR interval compared to younger patients (177 ms [160–200] in <70 years versus 189 ms [170–224] in 70–79 years versus 195 ms [175–233] in ≥80 years; *P* < 0.001). Median LVEF, at baseline, was similar between age categories (27% [25–34.8%] in <70 years versus 25% [20–34%] in 70–79 years versus 25% [25–35%] in ≥80 years; *P* = 0.23).

### Provision of guideline-directed medical therapy

There were no differences in the proportion of patients receiving a beta-blocker (94.8%, 92.6% and 90.0% in the <70, 70–79 and ≥ 80 cohorts, respectively; *P* = 0.26), however, younger patients were more often prescribed ACEi or angiotensin II receptor blockers (including sacubitril-valsaratan) (90.9%, 84.0% and 74.7% in the <70, 70–79 and ≥ 80 cohorts, respectively; *P* < 0.001). Relative to the maximum licensed doses of these agents, equivalent dosing of bisoprolol (6.2 mg ± 3.5, 5.2 mg ± 3.3 and 4.1 mg ± 2.9 in the <70, 70–79 and ≥ 80 cohorts, respectively; *P* < 0.001) and ramipril (6.8 mg ± 3.3, 6.3 mg ± 3.2 and 5.3 mg ± 3.4 in the <70, 70–79 and ≥ 80 cohorts, respectively; *P* = 0.0080) was lower for older patients. Those who were older were less likely to receive mineralocorticoid receptor antagonists (56.9%, 43.5% and 41.9% in the <70, 70–79 and ≥ 80 cohorts, respectively; *P* = 0.012). There were no differences in the proportion of patients receiving loop diuretics (67.9%, 73.3% and 76.5% in the <70, 70–79 and ≥ 80 cohorts, respectively; *P* = 0.22) and the mean dosing of these agents across age groups was also similar (78.4 mg ± 57.3, 72.5 mg ± 49.9 and 64.0 mg ± 44.4 in the <70, 70–79 and ≥ 80 cohorts, respectively; *P* = 0.16).

### Study procedures and complications

Older patients were less likely to be implanted with CRT-defibrillators compared to younger patients (<70 years [*n* = 80, 41.2%], 70–79 years [*n* = 82, 42.3%] and ≥ 80 years [*n* = 32, 16.5%]). The majority of patients underwent new device implantation, whereas 90 (15.7%) received their CRT on the background of a previous device; of those receiving upgrades, 75 were to CRT-pacemakers and 15 were to CRT-defibrillators. Procedural complications were uncommon with no differences across age groups: haematoma (*n* = 2, 0.3%), aortic dissection (*n* = 1, 0.2%), pneumothorax (*n* = 5, 0.9%) and device infection (*n* = 3, 0.5%). During follow-up, lead displacement occurred in 19 (3.3%) patients and of these, 15 (2.6%) patients required further intervention outside of the context of a generator replacement. Six (3.1%) patients implanted with CRT-defibrillators received inappropriate therapies from their device (Supplementary Table 1).

**Figure 2:**
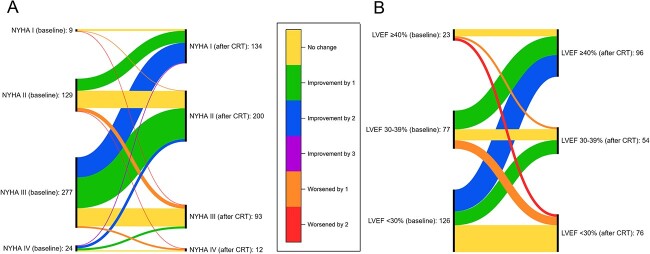
(A) Sankey diagram representing the change in NYHA class before and after CRT and (B) Sankey diagram representing the change in LVEF before and after CRT. NYHA, New York Heart Association; CRT, cardiac resynchronisation therapy; LVEF, left ventricular ejection fraction.

### Symptomatic improvement following cardiac resynchronisation therapy implantation

At follow up, data on NYHA classification was available for 439 (76.5%) participants. Of these, 267 (60.8%) improved at least one NYHA class (Figure 2A). Age was not associated with the rate of symptomatic improvement, either according to age category (59.8% versus 62.5% versus 59.3% responders in the <70, 70–79 and ≥80 cohorts, respectively; *P* > 0.05) or as a continuous variable (OR 1.01 per year [95% CI: 0.99, 1.03], *P* = 0.33). Worse symptoms as described by NYHA class at baseline were associated with greater odds of symptomatic response (III/IV; OR 5.32 [95% CI: 3.45, 8.23], *P* < 0.001). The prevalence of co-morbidities was similar between those that experienced symptomatic improvement and those that did not. Furthermore, although electrocardiographic findings of PR interval, QRS interval and left bundle branch block morphology were similar across both groups (with and without a symptomatic response), patients who experienced symptomatic improvement had a lower median LVEF (25% versus 28%; *P* = 0.015) at baseline. The provision of comprehensive guideline-directed medical therapy at baseline was not associated with symptomatic response following CRT implantation.

### Left ventricular function improvement following cardiac resynchronisation therapy implantation

Echocardiography data at follow up were available for 226 (39.4%) of patients who were alive at least six months following CRT implantation. Of these, 116 (51.3%) had an improvement of ≥10% LVEF on echocardiogram and 110 (48.7%) did not (Figure 2B). Age was not associated with improvements in LVEF following CRT (47.4% versus 53.6% versus 52.8% responders in the <70, 70–79 and ≥80 cohorts, respectively, *P* > 0.05), and neither were sex or more severe baseline symptoms (NYHA class III/IV). The prevalence of co-morbidities was similar across both groups. Electrocardiographic findings of PR interval, QRS interval, left bundle branch block morphology and echocardiographic LVEF were similar across those with and without improvement in LVEF, and there was no difference in baseline medical therapy in the two categories.

### Response to cardiac resynchronisation therapy according to either symptomatic improvement or change in LVEF

**Figure 3:**
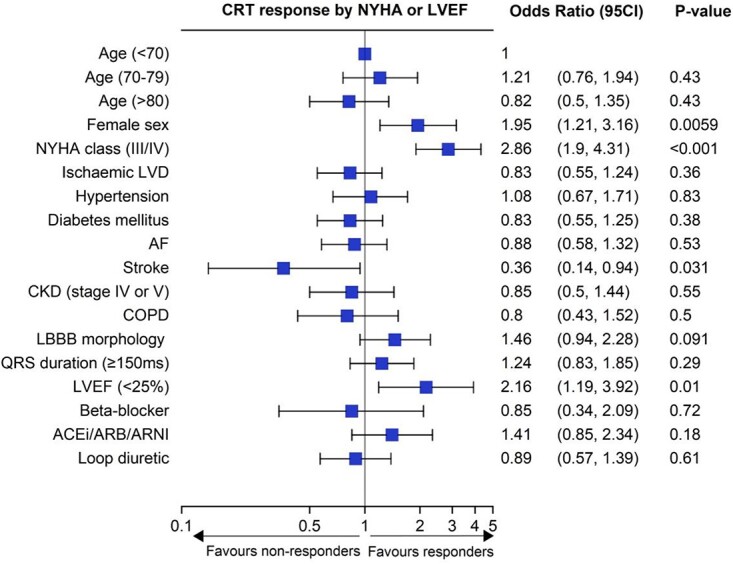
Forest plot depicting the predictors of CRT response. NYHA, New York Heart Association; LVD, left ventricular dysfunction; AF, atrial fibrillation; CKD, chronic kidney disease; COPD, chronic obstructive pulmonary disease; LBBB, left bundle branch block; ACEi, angiotensin receptor enzyme inhibitor; ARB, angiotensin receptor blocker; ARNI, angiotensin receptor neprilysin inhibitor.

Combining these measures of response, 466 (81.2%) participants had data on symptomatic status or echocardiography at follow up. Of these, 315 (67.6%) were classified as having ‘responded’ to CRT (Figure 3). In unadjusted analysis, age was not associated with response to CRT either as a categorical variable (67.2% versus 71.2% versus 62.6% responders in the <70, 70–79 and ≥ 80 cohorts, respectively, *P* = 0.43), or when described as a continuous variable (OR 1.00 per year [95% CI: 0.98, 1.02]; *P* =0. 80).

Predictors of response included female sex (OR 1.95 [95% CI: 1.21, 3.16], *P* = 0.0059), NYHA class III/IV symptoms (III/IV; OR 2.86 [95% CI: 1.90, 4.31], *P* < 0.001), and LVEF <25% (OR 2.16 [95% CI: 1.19, 3.92], *P* = 0.01). Other co-morbidities (ischaemic heart disease, hypertension, diabetes mellitus, atrial fibrillation, chronic kidney disease and chronic obstructive pulmonary disease) were not associated with response, although prior stroke was associated with lower odds of response (OR 0.36 [95% CI: 0.14, 0.94], *P* = 0.031). Although electrocardiographic findings of PR interval, QRS interval and left bundle branch block morphology were similar across both responders and non-responders, patients who improved had a lower median LVEF (25% versus 32%; *P* < 0.001) at baseline. There were no differences in the provision of guideline-directed medical therapy between responders and non-responders.

### Long-term outcomes

**Figure 4:**
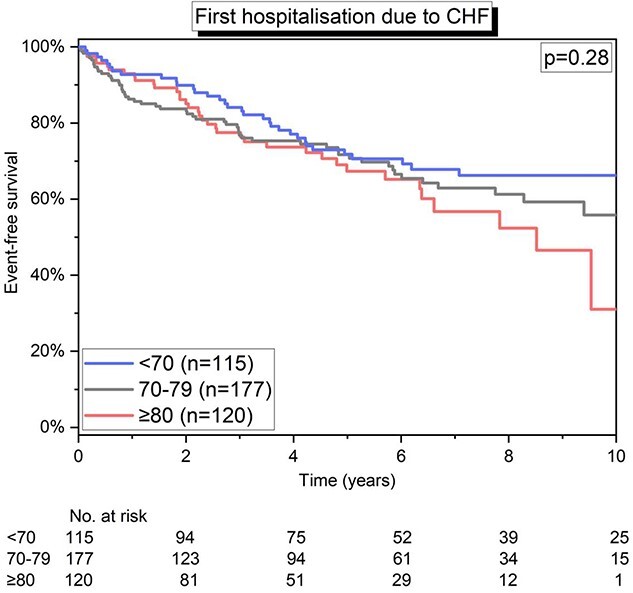
Kaplan–Meier curve showing the differences in time to first hospitalisation secondary to chronic heart failure (CHF) stratified by age groups.

During a median follow-up of 5.0 years (2.4–6.9), a total of 367 patients died. The estimated 1-, 5- and 10-year all-cause survival was 87.3% (84.6–90.1%), 54.9% (50.8–59.0%) and 25.8% (21.3–30.4%), respectively. After stratifying by age groups, estimated 10-year all-cause survival was less favourable for older patients (49.9% in <70 years; 23.9% in 70–79 years; and 6.9% in ≥80 years; *P* < 0.001). However, the time to first hospitalisation due to chronic heart failure was similar across age groups (*P* = 0.28; Figure 4).

Generator replacement was required for 91 (19.2%) patients during follow-up and was more often undertaken for younger patients (42 (32%), 42 (20.4%) and 7 (5.1%) for ages <70, 71–79 and ≥ 80 years; *P* < 0.001). Replacements were required around twice as frequently for CRT-defibrillator devices (*n* = 47, 28.3%) compared to CRT-pacemakers (*n* = 44, 14.2%). In patients who were still alive at study follow-up, generators were replaced more often in recipients with CRT-defibrillators (*n* = 28, 43.8%; median time to generator replacement 5.86 years) compared to those receiving CRT-pacemakers (*n* = 26, 29.9%; median time to generator replacement 7.61 years). Of patients receiving CRT-defibrillator devices, anti-tachycardia pacing and shocks occurred in 34 (20.5%) and 21 (12.7%) participants, respectively, and were more frequently observed in younger patients (20 [58.8%] and 16 [76.2%]) for <70 years, 12 [23.8%] and 5 [2.6%]) for 71–79 years, 2 [5.9%] and 0 [0%] for ≥80 years; *P* < 0.001 for both comparisons).

## Discussion

In this real-world cohort undergoing implantation of CRT for the management of HFrEF, we found that despite less optimal provision of guideline-directed medical therapy, older patients were as likely as younger patients to improve their symptomatic status or left ventricular systolic function following implantation. Although older patients had worse long-term survival, their risk of hospitalisation for worsening heart failure was similar.

### Age does not modify the treatment effect of cardiac resynchronisation therapy

Landmark trials demonstrating the prognostic and symptomatic benefits of CRT for the management of HFrEF have included populations with average ages between 63 and 67 years [4, 5, 12–15]. In clinical practice, the median age of patients newly referred with suspected heart failure is over 80 years [8], and so the external validity of data from these trials in contemporary populations is unclear. Moreover, these trials were conducted in an era before the more widespread implementation of guideline-directed medical therapies which could further attenuate the benefits of CRT [16].

Subsequently, large studies have looked at the effects of ageing on survival in older CRT recipients [17–19] and found that increasing age accounts for greater mortality during follow-up. However, older patients are more likely to have competing risks of non-cardiovascular co-morbidities than their younger counterparts [6], as shown in our data. Furthermore, observational studies in heart failure have shown that populations without heart failure matched for non-cardiovascular co-morbidities have similar actuarial survival [20–22]. The implication of this finding being that in the absence of other life limiting comorbidities, once adequately treated, even older patients can expect a reasonable prognosis when receiving contemporary pharmacological and device therapies for heart failure.

Accordingly, the assessment of response rate across age groups may be more useful. Several retrospective studies have reported response and survival data from populations receiving CRT using ≥75 years to define older patients [20, 23–27]. They found that despite greater mortality rates in older patients, response following CRT and the risk of subsequent hospitalisation for heart failure were similar. However, in contrast to our data, the representation of octogenarians in these studies was limited. Following inclusion of 173 patients aged ≥80 years that were followed-up for a median of 3.7 years, we found that the older patients had similar CRT response and time to first chronic heart failure hospitalisation compared to those that were younger, consistent with other studies that have included those aged ≥80 years [21, 22, 28].

### Defining ‘response’

Despite optimal guideline-directed medical therapy, chronic heart failure is characterised by a highly variable symptom burden and unpredictable disease trajectory [29]. One way of approaching response to CRT is to consider improvement in functional status or LV function. However, each correlates poorly with the other [30] and, compounded by the heterogeneity in the classification of response to CRT in prior studies [10, 31], true ‘response’ to CRT is difficult to define. Stable symptoms or a slower decline may be considered a meaningful outcome for some patients [32, 33]. Indeed, even ‘non-responders’ may experience a deterioration in their condition when CRT is temporarily deactivated [33]. Therefore, on an individual level, the absence of improvement following CRT does not always represent a lack of treatment effect and the results of studies such as ours should not be used to deselect groups of patients who do not ‘respond’ according to this binary definition. Hence, in a population of patients where a simple variable such as age is used to deselect patients for therapy, a comparison as we have done is reasonable to demonstrate that age alone should not be a discriminator.

In the present study of unselected consecutive patients, we observed a more favourable symptomatic response to CRT in those with worse functional status (NYHA III/IV; OR 2.86 [95% CI: 1.90, 4.31]) when compared to those with milder functional impairment (NYHA I/II). This finding contrasts with previous studies which used strict inclusion criteria with severe symptoms associated with less symptomatic improvement [34]. The lack of change in those with less severe symptoms is not surprising when one considers the four-category nature of the NYHA status and that NYHA class I implies no symptoms of breathlessness or fatigue, a state highly unlikely to be achieved in the presence of a chronic incurable condition. Hence a population consisting of patients with NYHA class II symptoms (modest symptoms on exercise) cannot improve further, whereas patients with more severe symptoms on mild activity (NYHA III) have the potential to improve. This pattern is also visible in the setting of remodelling where those with the worst LV function have the greatest magnitude of benefit.

### Complications

In our cohort complications occurred infrequently, consistent with previously reported data [26, 35]. Older patients more often received CRT-pacemakers and consequently were not at risk for inappropriate anti-tachycardia therapies. Furthermore, in those alive at follow-up, generators were replaced less often in recipients with CRT-pacemakers, likely reflecting their greater battery longevity. As a result, only 5% of patients ≥80 years underwent generator replacements.

### Limitations

This was a retrospective, non-randomised study and our findings should be interpreted in light of this. Analysis of CRT response was limited to patients who survived for a minimum of 6 months and attended for further clinical review. Differences in technique and experience between operators were not accounted for within our study, and the high rates of response and low complication rates might not be replicated in other datasets. Although left bundle branch block has been a historically consistent predictor of response [34], our dataset did not reflect this observation (OR 1.46 [95% CI 0.94, 2.28]), which may be due to the low prevalence of right bundle branch block morphology. Furthermore, we did not account for frailty, a known predictor of long-term mortality in those with chronic heart failure [36] which has been shown to predict non-response in patients receiving CRT [37–39]. However, given the lack of influence of age despite its clear association with frailty, this issue requires further exploration.

## Conclusion

In a large and representative cohort of people receiving CRT implantation, symptomatic or echocardiographic improvements were not influenced by age and the subsequent risk of hospitalisation for worsening heart failure was similar for older patients. Older people had worse symptomatic status and were more likely to have co-morbidities. Our findings support the provision of CRT in selected older patients.

## Supplementary Material

aa-23-1113-File007_afad222
