# Supplementary material for: Outcomes following cardiac resynchronisation therapy in older people
Source: Age Ageing. 2023 Nov 30;52(11):afad222. doi: 10.1093/ageing/afad222 (PMC10689176; doi:10.1093/ageing/afad222)
Supplement: aa-23-1113-File007_afad222 [file aa-23-1113-file007_afad222.docx]

Supplementary Files

Supplementary Figure 1: Histogram depicting the distribution of ages in the entire cohort.

Supplementary Table 1: Frequency of complications following cardiac resynchronisation therapy implantation.
